# THE RELATIONSHIP BETWEEN VOLUNTEERING AND SUBJECTIVE WELL-BEING AMONG OLDER ADULTS IN JAPAN

**DOI:** 10.1093/geroni/igae098.3439

**Published:** 2024-12-31

**Authors:** Koji Fujita, Yuri Yokoyama, Mariko Nishi, Hiroko Matsunaga, Yoshinori Fujiwara

**Affiliations:** Tokyo Metropolitan Institute for Geriatrics and Gerontology, Tokyo, Japan; Tokyo Metropolitan Institute for Geriatrics and Gerontology, Tokyo, Japan; Tokyo Metropolitan Institute for Geriatrics and Gerontology, Tokyo, Japan; Tokyo Metropolitan Institute for Geriatrics and Gerontology, Tokyo, Japan; Tokyo Metropolitan Institute for Geriatrics and Gerontology, Tokyo, Japan

## Abstract

The aim of this study is to examine the relationship between the frequency of volunteer activities among older adults and their subjective well-being. We mailed a questionnaire survey to 354 people aged 60 and over who belonged to volunteer organizations registered with a social welfare council within Saitama Prefecture. We received 235 responses for a response rate of 66.4%. Out of 215 participants, we excluded non-consenting and invalid responses, and subsequently analyzed the responses of 204 participants. We categorized these participants into three groups by frequency of volunteer activities: high activity, low activity, and non-activity. We performed a Kruskal-Wallis test with a multiple comparison test on the WHO–Five well-being index (range: 0-25 points). The high activity group accounted for 45.6% (n = 93), the low activity group for 35.8% (n = 73), and the non-activity group for 18.6% (n = 38). A significant difference was observed among the three groups in the WHO–5 (p=.013); the non-active group had the lowest mean score, while the high-activity group had the highest mean score. According to the multiple comparison test, this difference between the high and low activity groups was significant (p=.013). More frequent participation in volunteering among older adults is associated with higher subjective well-being. Volunteering may help maintain the physical and mental health of older adults and can reduce social isolation and loneliness. Therefore, participating in volunteer activities may help older adults to maintain and improve their mental health and subjective well-being.

